# Serum p‐tau217 Is a Prognostic Indicator of Cognitive Impairment in Idiopathic REM Sleep Behavior Disorder

**DOI:** 10.1002/ana.78109

**Published:** 2025-11-28

**Authors:** Shijun Yan, Anis Sahoo, Tanja Zerenner, Kenneth Marek, Michael Sommerauer, Wolfgang Oertel, Michele T. Hu, George K. Tofaris

**Affiliations:** ^1^ Nuffield Department of Clinical Neurosciences University of Oxford Oxford UK; ^2^ Kavli Institute for Nanoscience Discovery University of Oxford Oxford UK; ^3^ Population Health Sciences University of Bristol Bristol UK; ^4^ Institute for Neurodegenerative Disorders New Haven CT; ^5^ German Centre for Neurodegenerative Diseases (DZNE) Bonn Germany; ^6^ Centre of Neurology, Department of Parkinson, Sleep and Movement Disorders University of Bonn Bonn Germany; ^7^ Department of Neurology, University Hospital Cologne and Faculty of Medicine University of Cologne Cologne Germany; ^8^ Department of Neurology Philipps‐University Marburg Marburg Germany

## Abstract

**Objective:**

Assess the performance of serum phosphorylated tau 217 (p‐tau217) and neurofilament light chain (NfL) in predicting risk of cognitive impairment or phenoconversion to dementia in individuals with iRBD.

**Methods:**

We measured serum p‐tau217 and NfL levels by electrochemiluminescence across 4 polysomnographically confirmed iRBD cohorts (n = 300), including individuals who phenoconverted to Parkinson's disease (PD) (n = 51), dementia with Lewy bodies (DLB) (n = 22), and multiple system atrophy (MSA) (n = 5).

**Results:**

Serum p‐tau217 levels were increased in individuals with iRBD and cognitive impairment (CI) on testing defined as Montreal Cognitive Assessment <26 or subthreshold parkinsonism. p‐Tau217 differentiated individuals with iRBD who developed PD with CI (PD‐CI) or DLB from PD phenoconverters with normal cognition (area under curve [AUC] = 0.82; 95% confidence interval, 0.70–0.93) and from iRBD non‐phenoconverters with normal cognition (AUC = 0.83; 95% confidence interval, 0.77–0.89). NfL levels did not correlate with cognitive or motor scores and marginally improved p‐tau217 performance (AUC = 0.85; 95% confidence interval, 0.78–0.92), but were notably elevated in iRBD individuals who phenoconverted to MSA. Individuals with p‐tau217 in the top quartile were 8 times more likely to phenoconvert to PD‐CI or DLB compared to the bottom quartile (hazard ratio = 8.30; 95% confidence interval, 2.49–27.65).

**Interpretation:**

Serum p‐tau217, but not NfL, is a useful biomarker of cognitive impairment in iRBD that could be integrated into a multimodal prognostic indicator when stratifying risk of phenoconversion. ANN NEUROL 2026;99:912–921

Idiopathic rapid eye movement (REM) sleep behavior disorder (iRBD) is a well‐established prodrome of synucleinopathies, with many individuals eventually developing Parkinson's disease (PD), PD dementia (PDD), dementia with Lewy bodies (DLB), or multiple system atrophy (MSA).^1^ Although α‐synuclein aggregation is the main neuropathological hallmark, this pattern of pathology per se is not sufficient to explain the clinical heterogeneity observed across disease subtypes.^2^ It is, therefore, likely that co‐pathologies, especially of Alzheimer's disease (AD) type, contribute to the cognitive decline and/or influence the disease trajectory more broadly.

Postmortem and biomarker studies have identified tau pathology, especially in limbic and neocortical regions, in individuals with PDD and DLB.^3^, ^4^ Recent studies demonstrated that phosphorylated tau (p‐tau) isoform (p‐tau181) is increased in individuals with iRBD who go on to develop DLB.^5^ However, among tau species, plasma p‐tau217 has shown remarkable diagnostic specificity and sensitivity, outperforming other p‐tau isoforms in differentiating AD from non‐AD dementias and predicting amyloid‐β positivity across multiple cohorts.^6^, ^7^, ^8^ Despite these promising findings, the role of p‐tau217 in iRBD remains unexplored.

Serum neurofilament light (NfL) has gained attention as a sensitive, albeit generic marker of neurodegeneration. Serum or cerebrospinal fluid (CSF) NfL levels are increased in conditions with accelerated or diffuse neurodegeneration, such as motor neuron disease^9^ and dementias.^10^, ^11^ In synucleinopathies, NfL levels are most prominently increased in MSA, and to a lesser extent in DLB, but not in idiopathic PD.^12^, ^13^ However, in PD, serum NfL levels were shown to correlate with disease severity and motor deterioration.^14^ In a small prospective study, elevated baseline NfL levels in individuals with iRBD who later developed PD or DLB suggest a potential role for NfL as a risk‐marker of phenoconversion.^15^ The relevance of NfL has not been validated in larger iRBD cohorts and its clinical utility in prodromal synucleinopathies remains unclear.

Given the neuropathological heterogeneity that is inherent in synucleinopathies,^16^ reliance on a single biomarker may not be sufficient to provide adequate diagnostic or prognostic value. A multimodal strategy that integrates multiple serum biomarkers may improve risk stratification and guide the optimal timing of intervention with precision therapies. In this study, we assessed the performance of p‐tau217 vis‐à‐vis NfL in predicting risk of cognitive or motor impairment, as well as phenoconversion in individuals with iRBD.

## Methods

### Study Design

This is a retrospective cross‐sectional study of 4 cohorts that followed the Strengthening the Reporting of Observational Studies in Epidemiology (STROBE) reporting guidelines. The study profile is shown in Figure S1. Inclusion criteria for iRBD were confirmation by video‐assisted polysomnography and available serum samples. Participants were excluded if a secondary cause for RBD was present. Participants were recruited from July 2013 through February 2024, and samples were analyzed from May 2024 through April 2025. All patient data and samples were anonymized. The research ethics committees of the participating institutions approved the study. Participants provided written informed consent, and the protocols followed the principles of the Declaration of Helsinki. Oxford iRBD participants (n = 186) were recruited from the Discovery cohort.^17^ The Marburg iRBD cohort (n = 62) were recruited nationwide from the German RBD registry project and followed annually for at least 4 years.^18^ iRBD participants from Cologne (n = 37) were identified through structured population‐based screening process.^19^ The Parkinson's Progression Markers Initiative (PPMI) iRBD cohort (n = 15) were recruited across sites in the United States, Europe, or Israel (https://www.ppmi-info.org/). Subgroup analyses were performed to assess biomarker performance in individuals with additional prodromal markers. Where such results were available, we also assessed the association between biomarker levels and *APOE* genotype (n = 89) or phenoconversion (n = 78). Healthy controls without significant comorbidities or relevant family history were included from Oxford (n = 5) and Marburg (n = 2) as a reference group. The mean (standard deviation [SD]) time between blood sampling and last visit was 7.67 (2.99) years for participants with cognitive impairment (CI) and 6.71 (3.05) years for those who were cognitively normal (CN).

### Clinical Assessments

All subjects were assessed clinically as part of ongoing research programs. These included the updated Movement Disorder Society (MDS) prodromal criteria^20^ using MDS Unified Parkinson's Disease Rating Scale (UPDRS) part I and III, autonomic dysfunction (Scales for Outcomes in Parkinson's disease‐Autonomic Dysfunction), and the Montreal Cognitive Assessment (MoCA). CI on testing was defined as a MoCA score <26. Subthreshold parkinsonism (SP) was confirmed based on a MDS‐UPDRS‐III score >6, excluding postural and action tremor. Hyposmia was defined as identification scores <10th centile (score <10 for age >55 years and <13 for those age between 36 and 55) on Sniffin’ Sticks test, in the Oxford and Cologne cohorts; as a threshold/discrimination/identification (TDI) score ≤26 in the Marburg cohort; and as a ≤15th percentile score on the University of Pennsylvania Smell Identification Test (UPSIT), corrected for age and sex, in the PPMI cohort. Dopamine transporter (DaT) single photon emission computed tomography (SPECT) imaging was acquired using standard protocol. SPECT scans were classified as normal or abnormal based on descriptive reports provided by an expert nuclear medicine radiologist who was blinded to all clinical data other than age and sex, or based on previously described quantitative measures. Phenoconversion was defined based on respective diagnostic criteria for PD,^21^ DLB,^22^ and MSA.^23^

### Biomarker Measurements

Blood samples from all the centers were collected during the patient assessment and serum was isolated, aliquoted, and stored at −80°C until further use. All samples were sent on dry ice and processed in a blinded fashion at Oxford. Serum was the only biospecimen consistently available across all recruited participants in this retrospective cohort study. Serum p‐tau217 and NfL levels were measured using the Meso Scale Discovery (MSD, Rockville, MD, USA) electrochemiluminescence S‐PLEX human Tau kit (K151APFS‐2, MSD) and the R‐PLEX human NfL antibody set (F217X‐3, MSD), respectively, in 96‐well R‐Plex plates, following the manufacturer's protocols. All samples were visually inspected before analysis, and no hemolyzed samples were included. Undiluted serum was used for the p‐tau217 assay, and a 4‐fold dilution was applied for the NfL assay.

### Statistical Analyses

For comparisons of p‐tau217 and NfL levels, nonparametric statistical tests were used as the data were not normally distributed. The Kruskal‐Wallis 1‐way analysis of variance (ANOVA) with the Dunn's post hoc test was applied for comparisons involving 3 or more independent groups, and the Mann–Whitney *U* test was used for 2‐group comparisons. Receiver operating characteristic (ROC) curve analysis with 95% confidence intervals (2.5–97.5%) was performed to evaluate discriminatory performance. The optimal cut‐off point was determined by the Youden index, defined as the value maximizing sensitivity + specificity − 1. Age‐ and sex‐adjusted likelihood ratio (LR) for prodromal PD was calculated using the updated MDS research criteria.^20^ A positive LR was defined as a probability threshold of ≥80%. Age was compared using 1‐way ANOVA, and sex distribution using the χ^2^ test. Linear regression models adjusted for age and sex were used to assess associations between biomarkers and available prodromal markers. Logistic regression adjusted for age and sex was performed to determine the optimal biomarker combination for subgroup discrimination. Correlations between biomarkers and clinical measures were assessed using partial correlation adjusted for age and sex. Two‐tailed *p* values <0.05 were considered significant. Outliers were identified using the robust regression and outlier removal (ROUT) method. To study associations between biomarkers and risk of phenoconversion to PD‐CI or DLB, Cox regression was performed. Biomarkers were first considered individually while adjusting for age at time of sampling, sex, and storage time. Second, multivariable regression was fitted adjusting for age, sex, storage time, and mutually adjusting biomarkers for each other. Kaplan–Meier curves were plotted stratifying individuals according to biomarker levels using derived cut‐offs or quartiles. Hazard ratios (HR) with respect to the reference group were derived using Cox regression, and Schoenfeld residuals were assessed to check the proportional hazards assumption. Statistical analyses were performed using SPSS version 29.0 (IBM), Prism version 10.2.3 (GraphPad), and R version 4.1.1 (R Foundation).

## Results

### Demographic Characteristics

Among 307 participants included, the mean (SD) age was 66.59 (8.32) years, 268 were male (87.3%), and 39 were female (12.7%), as summarized in the Table 1. In the Oxford Discovery cohort, 191 participants (mean [SD] age, 66.70 [9.08] years; 164 male [85.9%] and 27 female [14.1%]) were included, and the mean (SD) time between blood sampling and last visit was 2.92 (2.70) years. In the Marburg cohort, 64 participants (67.75 [6.76] years; 60 male [93.8%] and 4 female [6.2%]) were included, and the mean (SD) time between blood sampling and last visit was 3.48 (2.09) years. In the Cologne cohort, 37 participants (66.59 [7.80] years; 31 male [83.8%] and 6 female [16.2%]) were included, and the mean (SD) time between blood sampling and last visit was 1.88 (1.35) years. In the PPMI cohort, 15 participants (70.80 [5.66] years; 13 male [86.7%] and 2 female [13.3%]) were included, and the mean (SD) time between blood sampling and last visit was 4.03 (3.54) years. Of the 307 participants, 299 self‐identified as White. Higher serum p‐tau217 was associated with older age and male sex, whereas no effects of age or sex were observed on serum NfL in iRBD (Table S1). There was a weak positive correlation between storage time and NfL levels (*ρ* = 0.14, *p* = 0.02) and a weak, borderline significant correlation with p‐tau217 levels (*ρ* = 0.11, *p* = 0.06), as shown in Figure S2. Antidepressant use had no effect on serum p‐tau217 or NfL levels (Fig S3).

**TABLE 1:** Demographic, Clinical, and Biomarker Characteristics of the iRBD Cohorts

Cohort	Characteristic	HC	iRBD non‐phenoconverters	PD‐CN phenoconverters	PD‐CI phenoconverters	DLB phenoconverters	MSA phenoconverters	P‐value^a^
Oxford Discovery	No of individuals	5	128	16	19	18	5	NA
	Sex, male/female	5/0	107/21	12/4	18/1	17/1	5/0	0.01
	Age, mean (SD), years	55.73 (2.35)	65.23 (9.32)	67.51 (6.52)	70.33 (7.16)	67.92 (8.08)	62.86 (9.09)	0.018
	Ethnicity, White, no (%)	5 (100.0)	123 (96.1)	16 (100.0)	17 (89.5)	18 (100.0)	5 (100.0)	0.04
	MoCA, mean (SD)	27.00 (2.35)	25.11 (3.08)	26.93 (1.79)	24.26 (2.33)	23.41 (3.30)	23.60 (2.19)	0.007
	Follow‐up after sampling, mean (SD), years	NA	3.47 (2.92)	3.46 (2.38)	2.81 (2.14)	3.29 (2.11)	2.14 (1.50)	0.74
	P‐tau217, median (IQR), pg/mL	0.35 (0.38)	0.67 (0.68)	0.57 (0.43)	1.05 (0.54)	1.33 (1.19)	0.36 (0.70)	<0.0001
	NfL, median (IQR), pg/mL	93.51 (206.77)	221.95 (357.63)	186.96 (288.87)	185.57 (243.22)	60.44 (117.17)	602.55 (90.34)	0.008
Marburg	No of individuals	2	45	12	2	3	NA	NA
	Sex, male/female	2/0	41/4	12/0	2/0	3/0	NA	NA
	Age, mean (SD), years	68.49 (—)	67.70 (7.28)	68.11 (5.52)	61.23 (—)	70.80 (3.16)	NA	NA
	Ethnicity, White, no (%)	2 (100.0)	45 (100.0)	12 (100.0)	2 (100.0)	3 (100.0)	NA	NA
	MoCA, mean (SD)	28.50 (—)	28.00 (1.70)	27.00 (2.78)	23.00 (—)	23.00 (2.83)	NA	NA
	Follow‐up after sampling, mean (SD), years	NA	3.56 (2.01)	3.27 (2.56)	1.77 (—)	4.29 (1.34)	NA	NA
	P‐tau217, median (IQR), pg/mL	0.62 (—)	0.50 (0.42)	0.49 (0.31)	1.04 (—)	1.12 (1.24)	NA	NA
	NfL, median (IQR), pg/mL	75.42 (—)	114.67 (247.18)	293.20 (73.81)	264.64 (—)	124.34 (215.83)	NA	NA
Cologne	No of individuals	NA	36	1	NA	NA	NA	NA
	Sex, male/female	NA	31/5	1/0	NA	NA	NA	NA
	Age, mean (SD), years	NA	66.29 (6.64)	77.38 (—)	NA	NA	NA	NA
	Ethnicity, White, no (%)	NA	36 (100.0)	1 (100.0)	NA	NA	NA	NA
	MoCA, mean (SD)	NA	27.08 (2.36)	27.00 (—)	NA	NA	NA	NA
	Follow‐up after sampling, mean (SD), years	NA	1.88 (1.37)	1.96 (—)	NA	NA	NA	NA
	P‐tau217, median (IQR), pg/mL	NA	0.26 (0.44)	0.14 (—)	NA	NA	NA	NA
	NfL, median (IQR), pg/mL	NA	93.92 (89.74)	62.60 (—)	NA	NA	NA	NA
PPMI	No of individuals	NA	13	1	NA	1	NA	NA
	Sex, male/female	NA	12/1	0/1	NA	1/0	NA	NA
	Age, mean (SD), years	NA	70.94 (5.94)	66.40 (—)	NA	73.20 (—)	NA	NA
	Ethnicity, White, no (%)	NA	12 (92.3)	1 (100.0)	NA	1 (100.0)	NA	NA
	MoCA, mean (SD)	NA	25.46 (4.74)	30.00 (—)	NA	30.00 (—)	NA	NA
	Follow‐up after sampling, mean (SD), years	NA	4.93 (3.28)	10.06 (—)	NA	10.18 (—)	NA	NA
	P‐tau217, median (IQR), pg/mL	NA	0.46 (0.82)	0.19 (—)	NA	0.60 (—)	NA	NA
	NfL, median (IQR), pg/mL	NA	63.75 (67.39)	724.51 (—)	NA	265.11 (—)	NA	NA

Data are number (%), mean (SD) or median (IQR), as appropriate. For groups with n < 3, only raw values or limited statistics are reported.

^a^ *p*‐Values show within‐cohort comparisons where group sizes were sufficient for statistical testing. One‐way ANOVA was used for age, MoCA, and follow‐up after sampling, the χ^2^ test for sex distribution, and the Kruskal‐Wallis test for p‐tau217 and NfL levels between groups.

ANOVA = analysis of variance; PD‐CI = Parkinson's disease with cognitive impairment on testing; DLB = dementia with Lewy bodies; HC = healthy controls; IQR = interquartile range; iRBD = idiopathic rapid eye movement sleep behavior disorder; MoCA = Montreal Cognitive Assessment, at the time of sampling; NA = not applicable; NfL = neurofilament light; PD‐CN = Parkinson's disease with normal cognition; MSA = multiple system atrophy; PPMI = Parkinson's Progression Markers Initiative; p‐tau217 = phosphorylated tau 217; SD = standard deviation.

### Serum p‐tau217 Is a Better than NfL in Predicting Cognitive and Motor Impairment in Individuals with iRBD

To evaluate early co‐pathology in individuals with iRBD, we measured serum p‐tau217 and NfL across 4 cohorts (Table 1). Although serum measurements are well established for NfL,^10^ absolute p‐tau217 concentrations may be lower in serum than plasma depending on the type of assay used even though values correlate well across the 2 matrices.^24^ Similarly, we validated our S‐PLEX MSD p‐tau217 assay, showing a high degree of correlation between our serum measurements in individuals with AD (n = 3), frontotemporal dementia (n = 2), or healthy controls (n = 3) and available matched plasma quantified by NULISA or Quanterix at a reference laboratory (Fig S4). We, then, assessed whether p‐tau217 or NfL levels are increased in iRBD individuals with ≥80% probability of being in the prodromal phase of PD as defined by the updated MDS prodromal research criteria (n = 250).^20^ Neither p‐tau217 nor NfL were per se reliable independent biomarkers of prodromal PD (Fig S5A, B). To determine whether any of the prodromal markers is individually associated with p‐tau217 or NfL, we performed multiple linear regression analysis in an iRBD subgroup where complete clinical data were available (n = 187). This analysis revealed that CI (β = 0.53 [standard error (SE) = 0.12]; *p* < 0.0001) and excessive daytime sleepiness (β = 0.34 [SE = 0.12]; *p* = 0.006) exhibit the strongest association with increased serum p‐tau217 levels (Table S2), whereas lower NfL levels were associated with olfactory dysfunction (β = −186.35 [SE = 70.21]; *p* = 0.009), as shown in Table S3.

**FIGURE 1:**
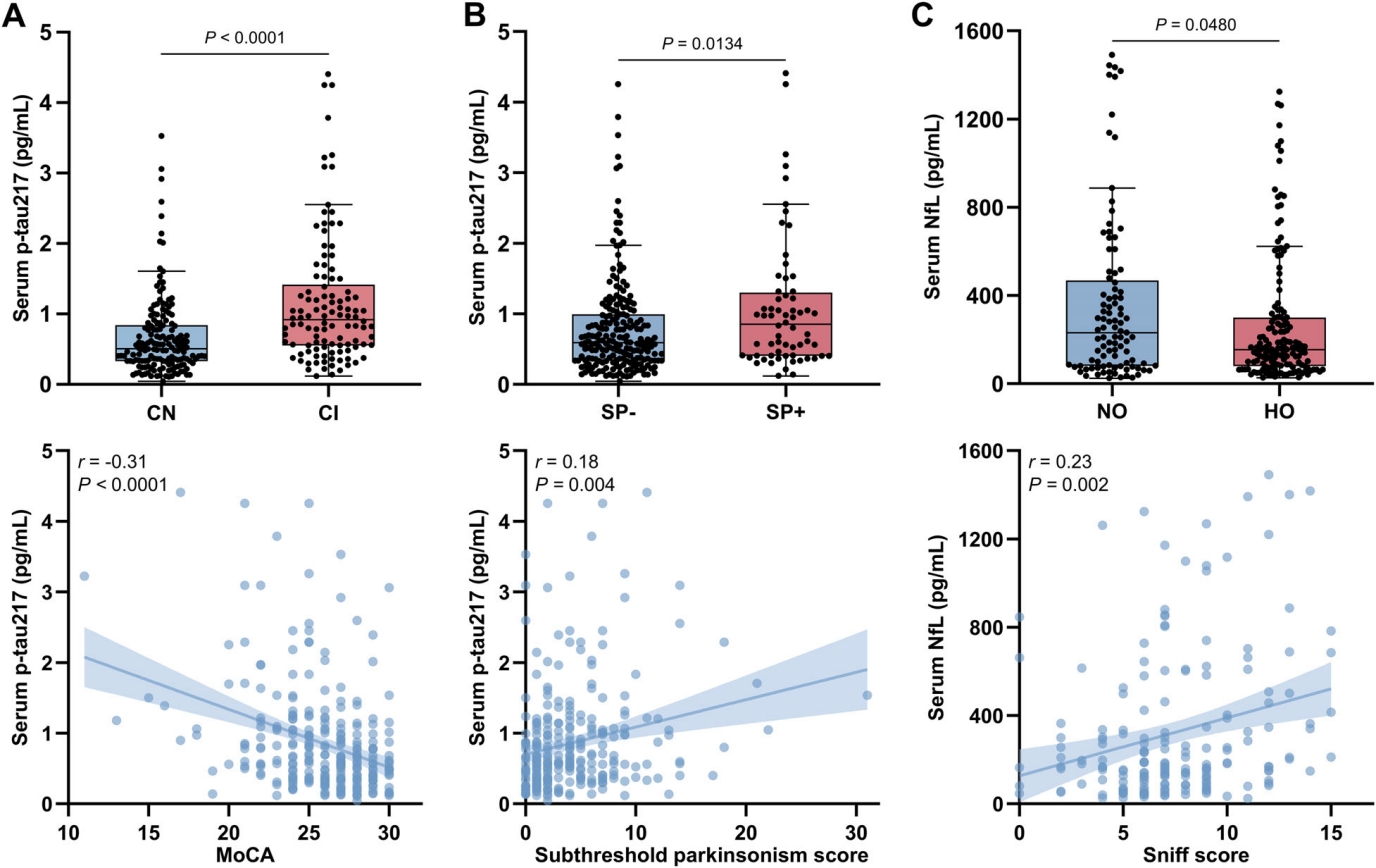
Serum phosphorylated tau 217 (p‐tau217) and neurofilament light (NfL) in idiopathic rapid eye movement sleep behavior disorder (iRBD) with additional prodromal markers. (A) Top: boxplot of p‐tau217 levels in individuals with iRBD and normal cognition (CN) (n = 176) or cognitive impairment (CI) on testing (n = 114). Bottom: scatter plot showing inverse partial correlation between p‐tau217 and MoCA scores, adjusted for age and sex. (B) Top: boxplot of p‐tau217 levels in iRBD individuals with subthreshold parkinsonism (SP+; n = 66) or without SP (SP−; n = 227), defined as Movement Disorder Society Unified Parkinson's Disease Rating Scale >6 excluding postural and action tremor. Bottom: scatter plot showing positive partial correlation between p‐tau217 and SP scores, adjusted for age and sex. (C) Top: boxplot of NfL levels in iRBD individuals with normosmia (NO) (n = 113) or hyposmia (HO) (n = 183). Bottom: scatter plot showing positive partial correlation between NfL and Sniffin’ Sticks test scores, adjusted for age and sex. Boxes represent the interquartile range from the 25th to 75th percentiles, the midline indicates the median, and whiskers and outliers are plotted using the Tukey method. Statistical significance was determined by the Mann–Whitney *U* test. Least squares regression lines with 95% confidence intervals are shown, with Pearson correlation coefficients and corresponding *p* values reported. [Color figure can be viewed at www.annalsofneurology.org]

In further subgroup analysis, iRBD individuals with CI, defined as a MoCA score <26 (iRBD‐CI; n = 114) had increased serum p‐tau217 levels (data shown as median ± interquartile range [IQR]: 0.92 ± 0.81pg/mL; *p* < 0.0001) compared to CN iRBD participants (iRBD‐CN; n = 176; 0.51 ± 0.50pg/mL) and p‐tau217 exhibited an inverse partial correlation with MoCA scores after adjusting for age and sex (*r* = −0.31, *p* < 0.001), as shown in Figure 1A. Interestingly, p‐tau217 levels were also increased in iRBD individuals with SP (SP+; n = 66; 0.85 ± 0.87pg/mL; *p* = 0.0134) relative to SP‐ individuals (n = 227; 0.58 ± 0.64pg/mL), and positively correlated with SP scores (*r* = 0.18, *p* = 0.004) as shown in Figure 1B. However, p‐tau217 levels were similar in iRBD individuals with an abnormal DaT SPECT (n = 47) compared to those with a normal DaT SPECT (n = 42), where this information was available (as shown in Fig S6A). ROC curve analyses of the aforementioned comparisons revealed modest discriminative power (Table S4). NfL levels did not differ between iRBD subgroups stratified by CI, SP, or DaT SPECT (Fig S6B–D). However, NfL levels were lower in iRBD individuals with hyposmia (n = 183; 154.22 ± 202.08pg/mL; *p* = 0.0480) compared to normosmic individuals (n = 113; 231.21 ± 376.94pg/mL), and positively correlated with Sniffin’ Sticks test scores (*r* = 0.23, *p* = 0.002) where these were available (Oxford and Cologne cohorts, n = 220), as shown in Figure 1C. No correlation was found between NfL and MoCA (*r* = −0.05, *p* = 0.48) or SP scores (*r* = 0.10, *p* = 0.88). Collectively, these diverse analyses identify p‐tau217, but not NfL as a useful adjunct biomarker when assessing individuals with iRBD at risk of cognitive impairment.

### Raised Serum p‐tau217 Levels Are Associated with Phenoconversion to PD with CI or DLB

**FIGURE 2:**
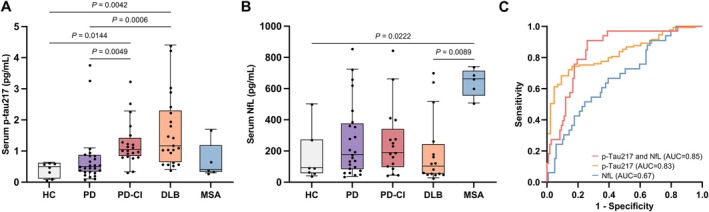
Serum phosphorylated tau 217 (p‐tau217) and neurofilament light (NfL) levels in individuals with idiopathic rapid eye movement sleep behavior disorder (iRBD) who phenoconverted. (A) Boxplot of p‐tau217 and (B) NfL levels in healthy controls (HC) (n = 7), phenoconverters to Parkinson's disease with normal cognition (PD) (n = 30), PD with cognitive impairment on testing (PD‐CI) (n = 21), dementia with Lewy bodies (DLB) (n = 22), or multiple system atrophy (MSA) (n = 5). (C) Logistic regression analysis controlled for age and sex evaluating the performance of p‐tau217, NfL, and their combination in distinguishing individuals who phenoconverted to PD‐CI or DLB from iRBD non‐phenoconverters with normal cognition. Boxes represent the interquartile range from the 25th to 75th percentiles, the midline indicates the median, and whiskers and outliers are plotted using the Tukey method. Statistical significance was determined by Kruskal‐Willis test (A and B). [Color figure can be viewed at www.annalsofneurology.org]

To further evaluate the clinical potential of serum biomarkers for dementia prediction, we identified 30 individuals with iRBD who phenoconverted to PD with normal cognition (PD), 21 to PD with CI on testing (PD‐CI), 22 to DLB, and 5 to MSA across cohorts. We found that serum p‐tau217 levels were higher in individuals who developed PD‐CI (1.05 ± 0.46pg/mL) and DLB (1.17 ± 1.45pg/mL) compared to PD phenoconverters (0.50 ± 0.50pg/mL) who exhibited levels similar to healthy controls (0.50 ± 0.38pg/mL), as shown in Figure 2A. In contrast, NfL levels were notably increased in those who phenoconverted to MSA (663.11 ± 85.74pg/mL), as shown in Figure 2B. There was no correlation (*r* = 0.06, *p* = 0.39) between p‐tau217 and NfL levels (Fig S7A).

In further logistic regression integrating age, sex, and biomarkers, we found that serum p‐tau217 separated those who phenoconverted to PD‐CI or DLB from non‐converters without CI, with an area under curve (AUC) of 0.83 (95% confidence interval, 0.77–0.89), sensitivity of 0.81 (95% confidence interval, 0.67–0.90), and specificity of 0.74 (95% confidence interval, 0.66–0.81) at the optimal threshold of 0.78pg/mL, derived by Youden's index when comparing iRBD participants with or without CI. In contrast, NfL only exhibited modest discriminative value (AUC = 0.67; 95% confidence interval, 0.57–0.77), and its combination with p‐tau217 only marginally improved the discrimination (AUC = 0.85; 95% confidence interval, 0.78–0.92) as shown in Figure 2C. p‐Tau217, but not NfL, also differentiated phenoconverters to PD‐CI and DLB from those who phenoconverted to PD with AUC of 0.82 (95% confidence interval, 0.70–0.93), as shown in Figure S7B. In those participants who phenoconverted to PD‐CI and DLB, there was no association between additional prodromal markers and p‐tau217 levels (Table S5).

### Time to Event Analysis

**FIGURE 3:**
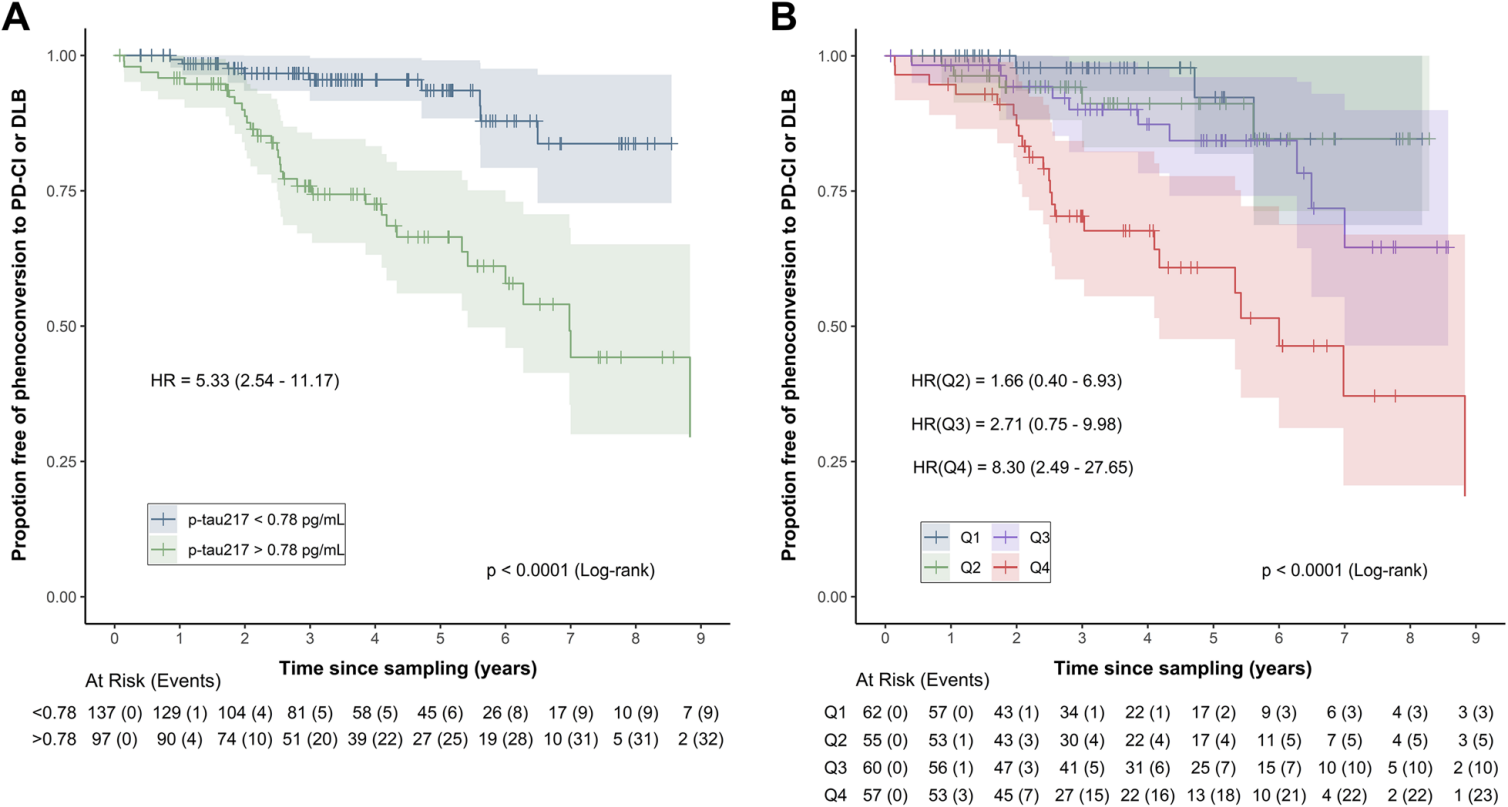
Kaplan–Meier survival curves for phenoconversion to Parkinson's disease (PD)‐cognitive impairment (CI) or dementia with Lewy bodies (DLB) as stratified by serum phosphorylated tau 217 (p‐tau217). (A) Individuals stratified by p‐tau217 levels above or below the optimal threshold (0.78pg/mL) for differentiating idiopathic rapid eye movement sleep behavior disorder (iRBD) individuals with and without cognitive impairment on testing (Montreal Cognitive Assessment [MoCA] <26). (B) Individuals stratified by p‐tau217 quartiles (Q1: <0.361, Q2: 0.361–0.605, Q3: 0.605–1.047, Q4: >1.047). Hazard ratios (HR) with 95% confidence intervals were derived from univariable Cox regression models, with patient group as a binary variable (A) or factor variable with quartiles (B). The vertical tick marks indicate censored observations. [Color figure can be viewed at www.annalsofneurology.org]

We then assessed the potential of serum p‐tau217 and/or NfL as prognostic biomarkers in iRBD, determining time to phenoconversion to PD‐CI or DLB. Stratification based on the optimal cut‐off (0.78 pg/mL) for differentiating iRBD‐CI from iRBD‐CN revealed that individuals with p‐tau217 >0.78 pg/mL were ~5 times (HR = 5.33; 95% CI, 2.54–11.57) more likely to phenoconvert to PD‐CI or DLB than individuals with p‐tau217 below this threshold (Fig 3A). Stratification based on quantiles showed that individuals with p‐tau217 in the top quartile were 8 times (HR = 8.30; 95% confidence interval, 2.49–27.65) more likely to phenoconvert to PD‐CI or DLB compared to the bottom quartile (Fig 3B). Cox regression considering p‐tau217 as a continuous variable showed that a 1pg/mL increase in p‐tau217 was associated with a ~2‐fold increased risk of phenoconversion to PD‐CI or DLB (Tables S6 and S7). In contrast, serum NfL levels were not associated with risk of phenoconversion to PD‐CI or DLB (Fig S8 and Tables S6 and S7). Adjusting for sample storage time did not alter these findings (Tables S8 and S9). Complementary time‐to‐event analyses for phenoconversion to DLB alone, using non‐phenoconverters without CI as the reference group, revealed a similar association (Fig S9).

### *APOE* Status and Biomarker Levels

*APOE* genotype was available for 89 iRBD participants from the Oxford Discovery and PPMI, including n = 37 *APOE* ε4 carriers (n = 36 heterozygous and n = 1 homozygous). Serum p‐tau217 levels were higher in the *APOE* ε4 carriers (n = 37; median ± IQR = 0.80 ± 0.69pg/mL) compared to the non‐carriers (n = 52; median ± IQR = 0.57 ± 0.56pg/mL), as shown in Figure S10, but the difference did not reach statistical significance (*p* = 0.07).

## Discussion

We showed that serum p‐tau217 levels are increased in iRBD individuals with CI on testing and correlate inversely with MoCA scores. Because blood p‐tau217 correlates with brain amyloid‐β deposition as assessed by positron emission tomography (PET),^25^ our data suggest that AD‐type co‐pathology is already detectable in individuals with prodromal synucleinopathy at risk of cognitive dysfunction or dementia. This observation is in line with growing neuropathological and biomarker‐based evidence that AD pathology contributes to the clinical heterogeneity of Lewy body diseases.^5^, ^26^ Increased p‐tau217 also correlated weakly with SP scores, but was not increased in iRBD individuals with abnormal DaT SPECT, suggesting that the effect on movement may be caused by AD‐type pathology in cortical regions of motor control. In agreement with this interpretation, slower gait speed even in cognitively unimpaired individuals is associated with higher mean cortical Aβ burden as assessed by PET^27^, ^28^ and gait disturbance often precedes the onset of both mild CI and AD.^29^ In addition, neuropathological studies in iRBD have not reported AD pathology in the brainstem.^30^

We found that p‐tau217 is most useful clinically in identifying those iRBD individuals who progress to CI or dementia phenotypes: participants with p‐tau217 >0.78pg/mL had 5 times higher risk compared to those with p‐tau217 below this threshold. These findings are consistent with recent reports of increased p‐tau217 levels in clinically diagnosed DLB^31^ and raised plasma p‐tau181 levels in iRBD individuals who developed DLB.^5^ Importantly, and in contrast to p‐tau181,^5^ our data demonstrate that p‐tau217 in iRBD also identifies PD‐CI in iRBD. Interestingly, we observed that p‐tau217 levels even associate with excessive daytime sleepiness, which was previously reported to correlate with AD‐type pathology as quantified by PET or CSF biomarkers.^32^, ^33^ Therefore, greater subjective daytime sleepiness in iRBD may be a harbinger of progression to dementia. Although the optimal p‐tau species in prodromal α‐synucleinopathy will require vis‐à‐vis comparisons in longitudinal cohorts, p‐tau217 is known to exhibit superior specificity compared to p‐tau181, at least in prodromal AD.^8^ The modest performance of serum p‐tau217 in distinguishing iRBD‐CI from iRBD‐CN reflects at least partly our stratification, which relied only on basic cognitive testing (MoCA <26) that was consistently available across all recruitment sites. Incorporation of additional measures of cognitive function is likely to improve this differentiation. Nevertheless, our findings show that increased serum p‐tau217 levels in iRBD is an early indicator of even subtle cognitive dysfunction.

Serum p‐tau217 has a comparable diagnostic accuracy to plasma p‐tau217 across most assay platforms.^24^ Nevertheless, it is important to note that absolute biomarker concentrations and optimal clinical cut‐off values have not yet been fully established across assays or biomatrices (serum vs plasma). They are also likely to differ across clinical indications or be influenced by distinct genetic modifiers. For example, although we found some evidence that serum p‐tau217 may be increased in *APOE4* carriers (*p* = 0.07), a larger sample size is required to further evaluate this association, which may not be as strong as the one reported in AD.^34^ Plasma p‐tau181 was previously shown not to be affected by *APOE* status in iRBD.^5^

Despite the promise of serum NfL in prodromal AD,^10^ this biomarker was not increased in iRBD participants with CI nor did it associate with phenoconversion to DLB or PD‐CI. It is noteworthy that serum p‐tau217 and NfL levels did not correlate, suggesting that AD‐type pathology may not be the main driver of neurodegeneration in iRBD as reflected by NfL levels. Although limited by numbers, our data suggest that markedly elevated NfL levels in iRBD most likely signify progression to MSA, which is consistent with prior findings of increased NfL levels in clinically diagnosed MSA cases.^12^ Therefore, NfL measurements in iRBD could be most useful clinically as a prognostic indicator in this context. The association between olfactory dysfunction and lower NfL levels requires further investigation, but it suggests that hyposmic individuals with iRBD may have a disease subtype that is associated with less neuronal degeneration.

There are limitations to our findings. First, phenoconversion is based on clinical diagnosis without neuropathological examination, and it is possible that some of these diagnoses will be incorrect. Second, although the correlation between p‐tau217 blood levels and brain Aβ burden is well established,^25^ amyloid PET were not available, limiting the mechanistic interpretation of the underpinning pathology in iRBD individuals with increased p‐tau217. Third, although we analyzed the largest iRBD group to date for p‐tau blood biomarker assessment, the number of individuals who phenoconverted was relatively small, which precludes definitive estimation of sensitivity and specificity. The serum cut‐off value used reflects relative risk within the iRBD cohort and further studies in healthy asymptomatic populations are needed to define reference ranges for clinical application. iRBD is much more common in men^35^ and the preponderance of male participants in the cohorts requires caution when extending any conclusions to females. Additionally, almost all study participants self‐identified as White and our results may not generalize to other racial or ethnic populations.

In conclusion, our findings identify serum p‐tau217 as a prognostic indicator for cognitive impairment in iRBD and a potential stratification biomarker when designing clinical trials for precision disease‐modifying therapies in this condition. Validation of these results in longitudinal cohorts incorporating additional co‐pathology biomarkers such as L1CAM+ extracellular vesicle,^36^ or seed amplification assays^37^ for α‐synuclein, will provide deeper insights into the temporal dynamics of pathological trajectories relative to clinical progression.

## Author Contributions

S.Y., A.S., and G.K.T, contributed to the conception and design of the study. S.Y., A.S., T.Z., K.M., M.S., W.O., M.T.H., and G.K.T., contributed to the acquisition and analysis of data. S.Y., and G.K.T., contributed to drafting the text or preparing the figures.

## Potential Conflicts of Interest

Nothing to report.

## Supporting information


**Data S1.** Supporting Information.

## Data Availability

Anonymized individual participant data and the study protocol will be shared with qualified parties on request to the corresponding author (G.K.T.).
