# Supplementary material for: Serum p‐tau217 Is a Prognostic Indicator of Cognitive Impairment in Idiopathic REM Sleep Behavior Disorder
Source: Ann Neurol. 2025 Nov 28;99(4):912–21. doi: 10.1002/ana.78109 (PMC13011788; doi:10.1002/ana.78109)
Supplement: Supplementary file 1 — Data S1. Supporting Information. [file ANA-99-912-s001.pdf]

Supporting Information

Serum p-tau217 is a prognostic indicator of cognitive impairment in idiopathic REM sleep behaviour disorder

Supplementary Tables 3

Supplementary Table S1: Influence of age and sex on serum p-tau217 or NfL........ 3

Supplementary Table S2: Association between serum p-tau217 levels and prodromal markers in iRBD........ 4

Supplementary Table S3: Association between NfL levels and prodromal markers in iRBD........ 5

Supplementary Table S4: ROC analyses of p-tau217 in the iRBD subgroup comparisons........ 6

Supplementary Table S5: Association between serum p-tau217 levels and prodromal markers in PD-CI and DLB phenoconverters 7

Supplementary Table S6: Cox regression for time to phenoconversion to PD-CI on testing or DLB 8

Supplementary Table S7: Mutually adjusted Cox regression for time to phenoconversion to PD-CI on testing or DLB........ 8

Supplementary Table S8: Cox regression for time to phenoconversion to PD-CI on testing or DLB adjusted for sample storage time........ 9

Supplementary Table S9: Mutually adjusted Cox regression for time to phenoconversion to PD-CI on testing or DLB adjusted for storage time 9

Supplementary Figures........ 10

Supplementary Figure S1: Study profile 10

Supplementary Figure S2: Correlations between storage time and biomarker levels 11

Supplementary Figure S3: Serum p-tau217 and NfL levels in iRBD stratified by antidepressant use 12

Supplementary Figure S4: Correlations between p-tau217 levels in matched serum measured by MSD and plasma measured by NULISA or Quanterix ALZpath 13

Supplementary Figure S5: Serum p-tau217 and NfL levels in iRBD stratified by likelihood ratio of prodromal PD........ 14

Supplementary Figure S6: Serum p-tau217 and NfL levels in iRBD with additional prodromal marker........ 15

Supplementary Figure S7: Correlation between serum p-tau217 and NfL levels, and logistic regression analysis of their performance in distinguishing phenoconversion to PD-CI on testing or DLB from PD with normal cognition 16
Supplementary Figure S8: Kaplan-Meier survival curves for phenoconversion to PD-CI on testing or DLB with subjects stratified by NfL 17
Supplementary Figure S9: Kaplan-Meier survival curves for phenoconversion to DLB with subjects stratified by p-tau217 using non-phenoconverters without CI as the reference group 18
Supplementary Figure S10: Serum p-tau217 and NfL levels in iRBD stratified by *APOE* status ... 19

Supplementary Tables

Supplementary Table S1：Influence of age and sex on serum p-tau217 or NfL

Serum p-tau217				Serum NfL			
**Variable**	**β**	**SE**	**P-value**	**Variable**	**β**	**SE**	**P-value**
Intercept	-0.025	0.072	0.73	Intercept	2.463	0.232	<0.0001
Age	0.003	0.001	0.001	Age	-0.003	0.003	0.33
Sex [Male]	0.053	0.025	0.04	Sex [Male]	0.129	0.079	0.10
Model R-square	0.05			Model R-square	0.02		
Model P-value	0.0005			Model P-value	0.15		

Multiple linear regression within iRBD group reveal an association between p-tau217 and sex or age using the equation $\log(\text{p-tau217}+1) = \beta 0 + (\beta 1 \times \text{Age}) + (\beta 2 \times \text{Sex}) + \varepsilon$, while no significant associations with age or sex were observed for serum NfL using the equation $\log(\text{NfL}) = \beta 0 + (\beta 1 \times \text{Age}) + (\beta 2 \times \text{Sex}) + \varepsilon$. Abbreviations: SE = standard error.

Supplementary Table S2: Association between serum p-tau217 levels and prodromal markers in iRBD

Variable	β	SE	P-value
Intercept	-0.17	0.46	0.71
Age	0.01	0.007	0.08
Sex [Male]	0.09	0.17	0.61
Cognitive impairment [Yes]	0.53	0.12	<0.0001
Subthreshold parkinsonism [Positive]	0.13	0.14	0.33
Olfaction [Abnormal]	-0.09	0.14	0.51
Excessive daytime sleepiness [Yes]	0.34	0.12	0.006
Constipation [Yes]	-0.08	0.12	0.51
Urinary dysfunction [Yes]	-0.17	0.12	0.15
Symptomatic OH [Yes]	0.12	0.15	0.41
Depression [Yes]	-0.21	0.13	0.12

Multiple linear regression modelling identified mild cognitive impairment based on MoCA < 26 and excessive daytime sleepiness as having the strongest association with increased serum p-tau217 levels. Abbreviations: OH = orthostatic hypotension.

Supplementary Table S3: Association between NfL levels and prodromal markers in iRBD

Variable	β	SE	P-value
Intercept	411.91	258.90	0.11
Age	2.03	3.63	0.58
Sex [Male]	-76.86	78.03	0.33
Cognitive impairment [Yes]	17.13	61.44	0.78
Subthreshold parkinsonism [Positive]	10.22	66.78	0.88
Olfaction [Abnormal]	-186.35	70.21	0.009
Excessive daytime sleepiness [Yes]	-65.39	59.58	0.27
Constipation [Yes]	-5.41	56.69	0.92
Urinary dysfunction [Yes]	-79.09	56.02	0.16
Symptomatic OH [Yes]	-56.07	75.09	0.46
Depression [Yes]	-37.97	61.97	0.54

Multiple linear regression modelling identified hyposmia as having the strongest association with lower serum NfL levels. Abbreviations: OH = orthostatic hypotension.

Supplementary Table S4: ROC analyses of p-tau217 in the iRBD subgroup comparisons

	**CN vs CI**	**SP- vs SP+**
AUC (95% CI)	0.70 (0.64 – 0.76)	0.60 (0.52 – 0.68)
Optimal threshold (pg/mL)[a]	0.78	0.94
Sensitivity (95% CI)	0.62 (0.52 – 0.70)	0.48 (0.37 – 0.60)
Specificity (95% CI)	0.72 (0.65 – 0.79)	0.73 (0.66 – 0.78)

Abbreviations: AUC = area under the receiver operating characteristic curve; CI = cognitive impairment; CN = cognitively normal; SP = subthreshold parkinsonism

[a] Optimal threshold was determined by the Youden's index for each comparison and the respective sensitivity and specificity were reported.

Supplementary Table S5: Association between serum p-tau217 levels and prodromal markers in PD-CI and DLB phenoconverters

Variable	β	SE	P-value
Intercept	0.98	2.60	0.71
Age	0.004	0.03	0.89
Sex [Male]	0.04	0.90	0.97
Cognitive impairment [Yes]	0.24	0.50	0.63
Subthreshold parkinsonism [Positive]	0.06	0.47	0.90
Olfaction [Abnormal]	0.09	0.53	0.87
Excessive daytime sleepiness [Yes]	0.07	0.54	0.89
Constipation [Yes]	-0.35	0.45	0.44
Urinary dysfunction [Yes]	-0.01	0.43	0.98
Symptomatic OH [Yes]	0.49	0.58	0.41
Depression [Yes]	-0.10	0.43	0.82

Multiple linear regression modelling identified no association between individual prodromal markers and serum p-tau217 levels in those iRBD participants who phenoconverted to PD-CI and DLB. Abbreviations: OH = orthostatic hypotension.

Supplementary Table S6: Cox regression for time to phenoconversion to PD-CI on testing or DLB

Variable	HR (95% CI)	P-value	N (obs)	N (events)
p-tau217 (pg/mL)	1.92 (1.47 - 2.51)	2.04×10^{-6}	234	42
NfL (10 pg/mL)	1.00 (0.98 – 1.01)	0.56	210	32

Cox proportional hazards models models were fitted for each marker and adjusted for age and sex; Hazard ratios for NfL reflects the increase in phenoconversion risk associated with a 10 pg/mL increase in NfL. Abbreviations: HR = hazard ratio; obs = observations.

Supplementary Table S7: Mutually adjusted Cox regression for time to phenoconversion to PD-CI on testing or DLB

Variable	HR (95% CI)	P-value
p-tau217 [pg/mL]	2.12 (1.43 – 3.13)	0.0002
NfL [10 pg/mL]	0.99 (0.98 - 1.01)	0.45

Cox proportional hazards models were fitted including both serum p-tau217 and NfL as covariates, mutually adjusted and additionally controlled for age and sex. Model was fitted to n = 194 observations including 32 cases who phenoconverted to PD-CI on testing or DLB. Abbreviations: HR = hazard ratio.

Supplementary Table S8: Cox regression for time to phenoconversion to PD-CI on testing or DLB adjusted for sample storage time

Variable	HR (95%-CI)	P-value	N (obs)	N (events)
p-tau217 [pg/mL]	1.97 (1.50- 2.57)	7.6×10^{-7}	234	42
NfL [10 pg/mL]	1.00 (0.98 – 1.01)	0.61	210	32

Individual models were fitted for each marker and adjusted for age, sex, and sample storage time; Hazard ratios (HR) for NfL reflects the increase in phenoconversion risk associated with a 10 pg/ml increase in NfL.

Supplementary Table S9: Mutually adjusted Cox regression for time to phenoconversion to PD-CI on testing or DLB adjusted for storage time

Variable	HR (95%-CI)	P-value
p-tau217 [pg/mL]	2.10 (1.44 – 3.06)	0.0001
NfL [10 pg/mL]	1.00 (0.98 - 1.01)	0.56

Cox proportional hazards models were fitted including both serum p-tau217 and NfL as covariates, mutually adjusted and additionally controlled for age, sex, and storage time. Model was fitted to n =194 observations including 32 cases of phenoconversion to PD-MCI or DLB.

Supplementary Figures

Supplementary Figure S1: Study profile

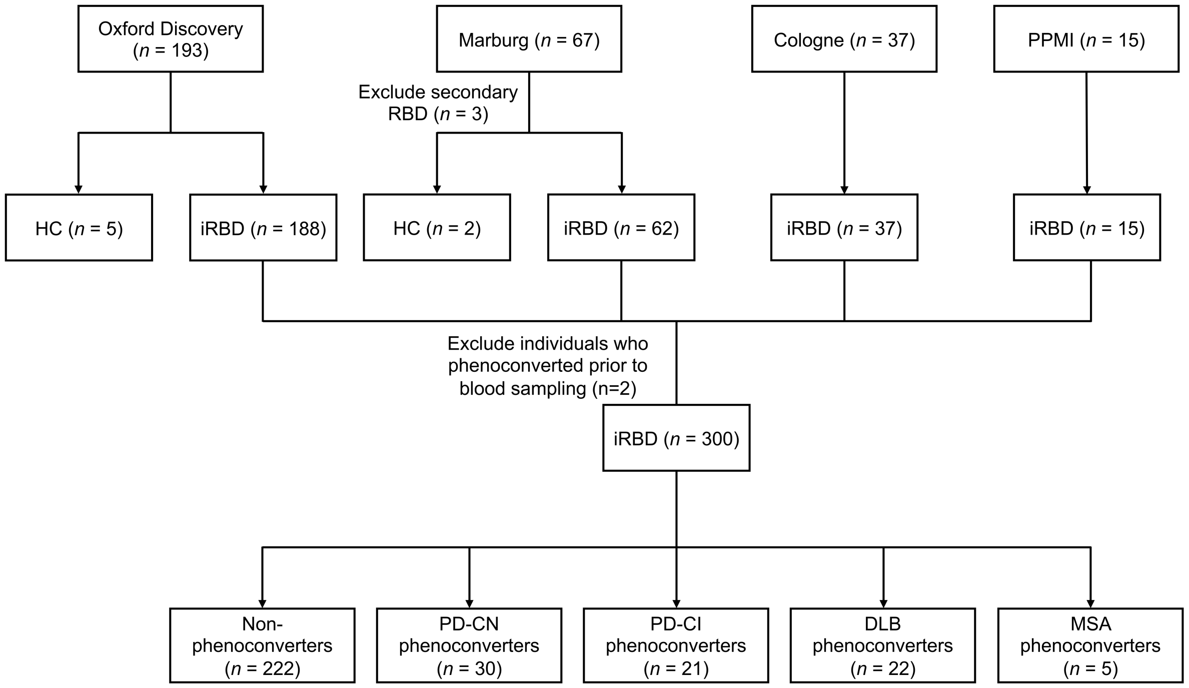

Supplementary Figure S2: Correlations between storage time and biomarker levels

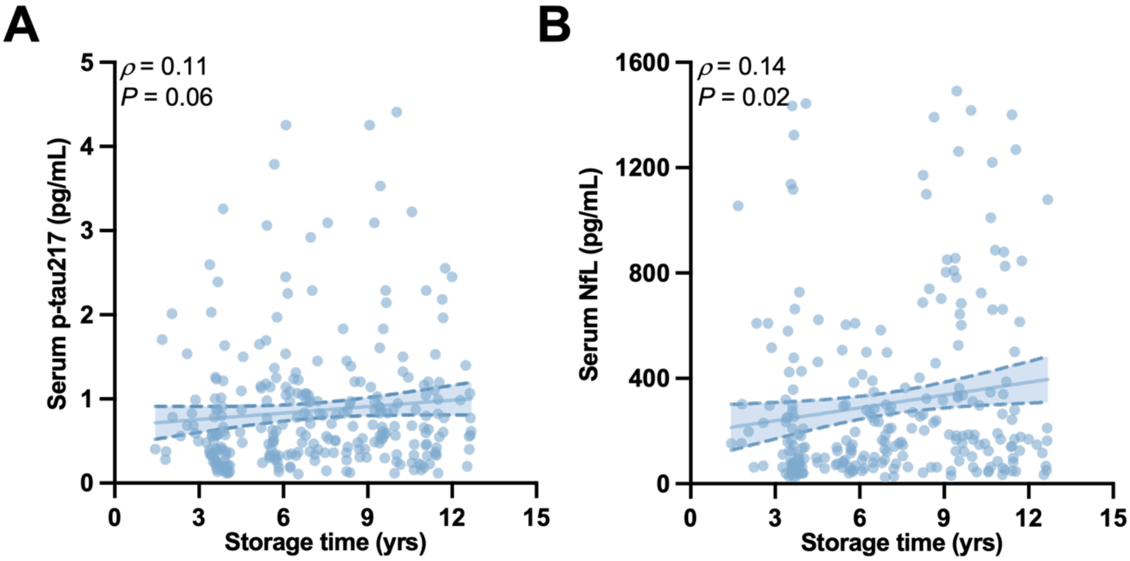

(**A**) Scatter plot showing no significant partial correlation between storage time and serum p-tau217 levels. (**B**) Scatter plot showing a weak positive partial correlation between storage time and serum NfL levels. Least squares regression line with 95% confidence interval is shown. Spearman correlation coefficient and corresponding P value are reported.

Supplementary Figure S3: Serum p-tau217 and NfL levels in iRBD stratified by antidepressant use

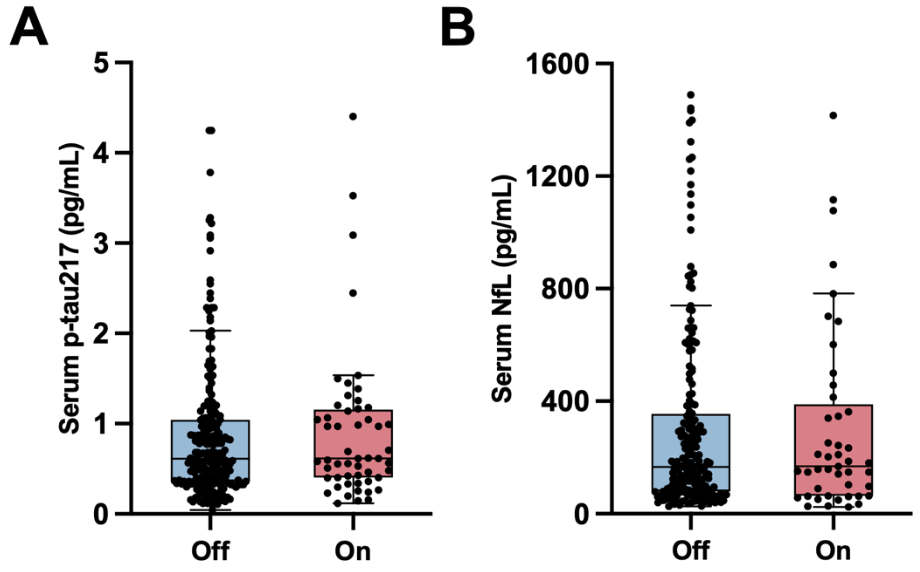

(**A**) Boxplot of p-tau217 and (**B**) NfL measurements in iRBD subjects off (n = 244) or on antidepressant (n = 56). Boxes represent the interquartile range from the 25th to 75th percentiles, the midline indicates the median, and whiskers and outliers are plotted using the Tukey method. Statistical comparisons were performed using the Mann–Whitney U test.

Supplementary Figure S4: Correlations between p-tau217 levels in serum measured by MSD and matched plasma measured by NULISA or Quanterix ALZpath at a reference laboratory

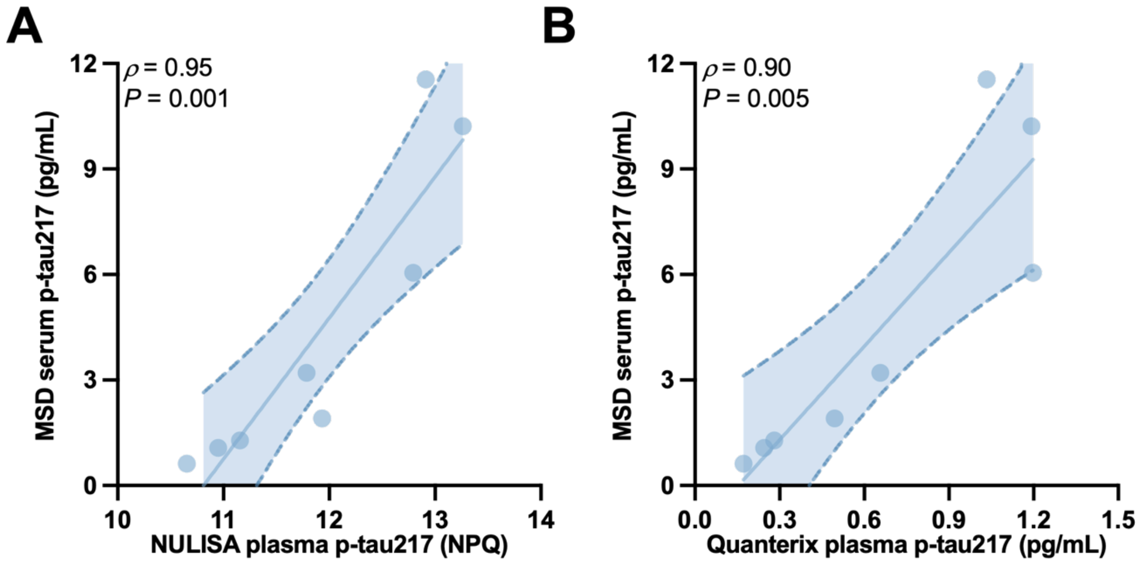

Scatter plot showing strong correlations between serum p-tau217 levels measured by the S-PLEX MSD assay and matched plasma p-tau217 levels measured by **(A)** NULISA or (**B**) Quanterix ALZpath. Least squares regression line with 95% confidence interval is shown. Spearman correlation coefficient and corresponding P value are reported.

Supplementary Figure S5: Serum p-tau217 and NfL levels in iRBD stratified by likelihood ratio of prodromal PD

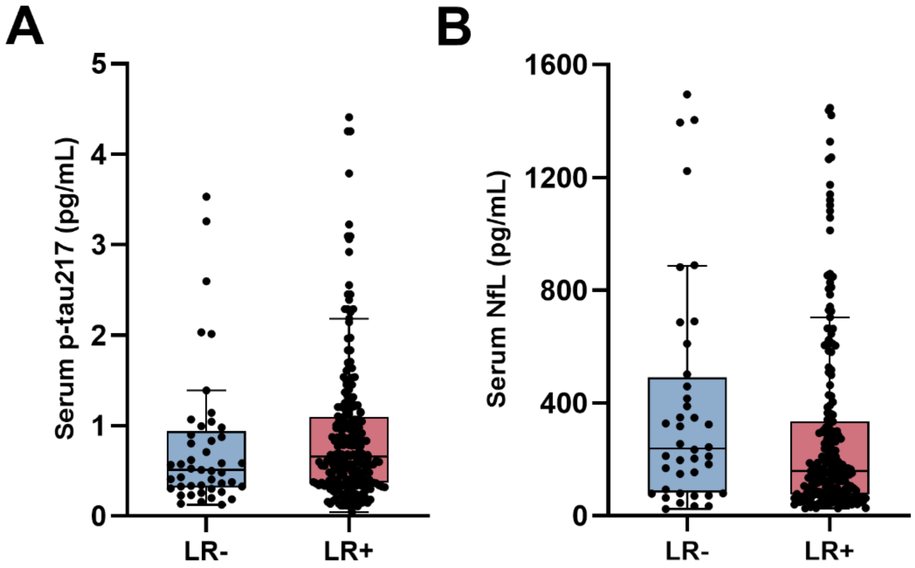

(**A**) Boxplot of p-tau217 and (**B**) NfL measurements in iRBD subjects with a positive likelihood ratio (LR+; n = 250) or negative likelihood ratio (LR-; n = 50) of prodromal PD, based on the updated Movement Disorder Society prodromal research criteria. Boxes represent the interquartile range from the 25th to 75th percentiles, the midline indicates the median, and whiskers and outliers are plotted using the Tukey method. Statistical comparisons were performed using the Mann–Whitney U test.

Supplementary Figure S6: Serum p-tau217 and NfL levels in iRBD with additional prodromal marker

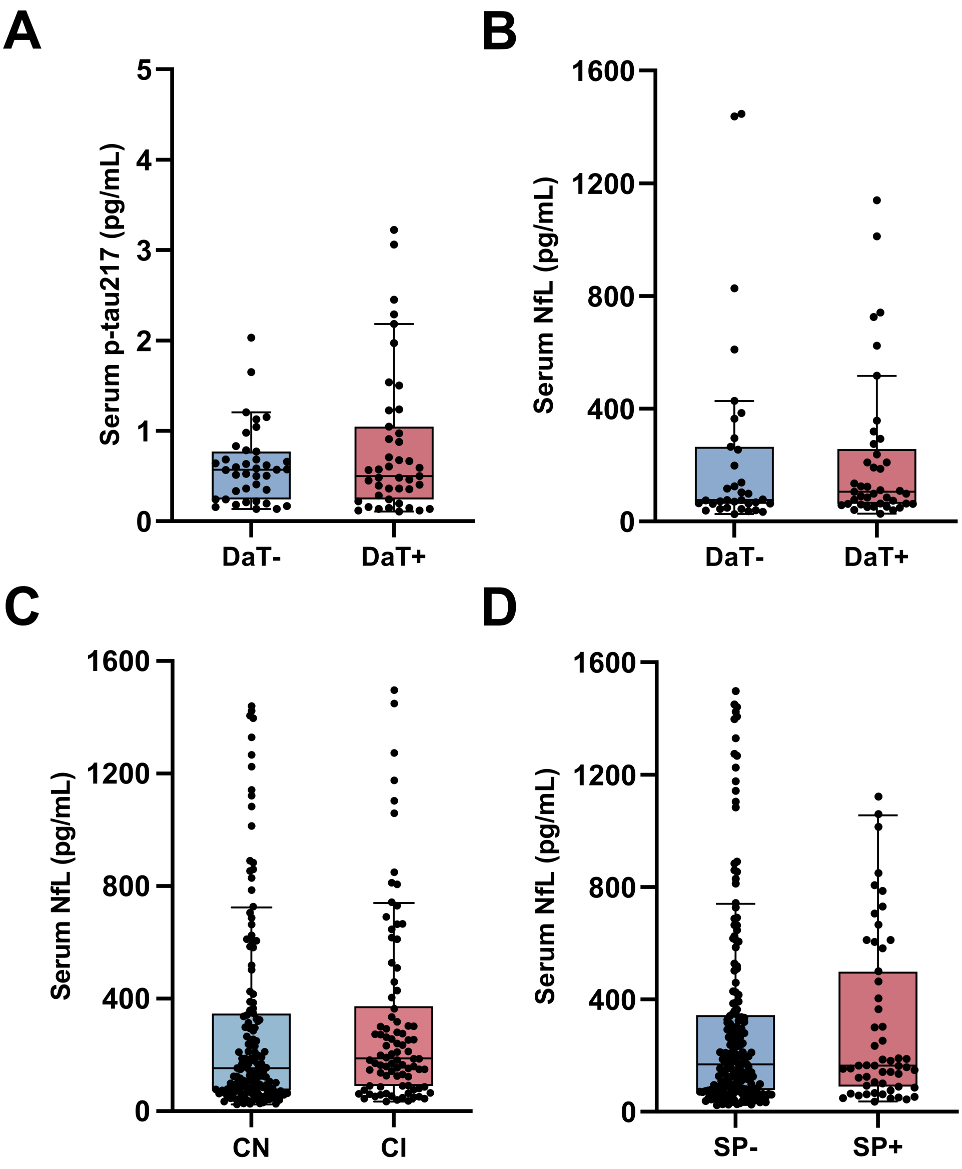

(**A**) Boxplot of p-tau217 and (**B**) NfL measurements in iRBD subjects with a negative/normal (DaT-; *n* = 42) or positive/abnormal (DaT+; *n* = 47) DAT SPECT. (**C**) Boxplot of NfL measurements in iRBD subjects with normal cognition (CN; *n* = 176) or cognitive impairment (CI; *n* = 114). (**D**) Boxplot of NfL levels in iRBD individuals with (SP+; *n* = 66) or without (SP-; *n* = 227) subthreshold parkinsonism (SP), defined as MDS-UPDRS-III > 6 excluding postural and action tremor. Boxes represent the interquartile range from the 25th to 75th percentiles, the midline indicates the median, and whiskers and outliers are plotted using the Tukey method. Statistical comparisons were performed using the Mann–Whitney U test.

Supplementary Figure S7: Correlation between serum p-tau217 and NfL levels, and logistic regression analysis of their performance in distinguishing phenoconversion to PD-CI or DLB from PD with normal cognition

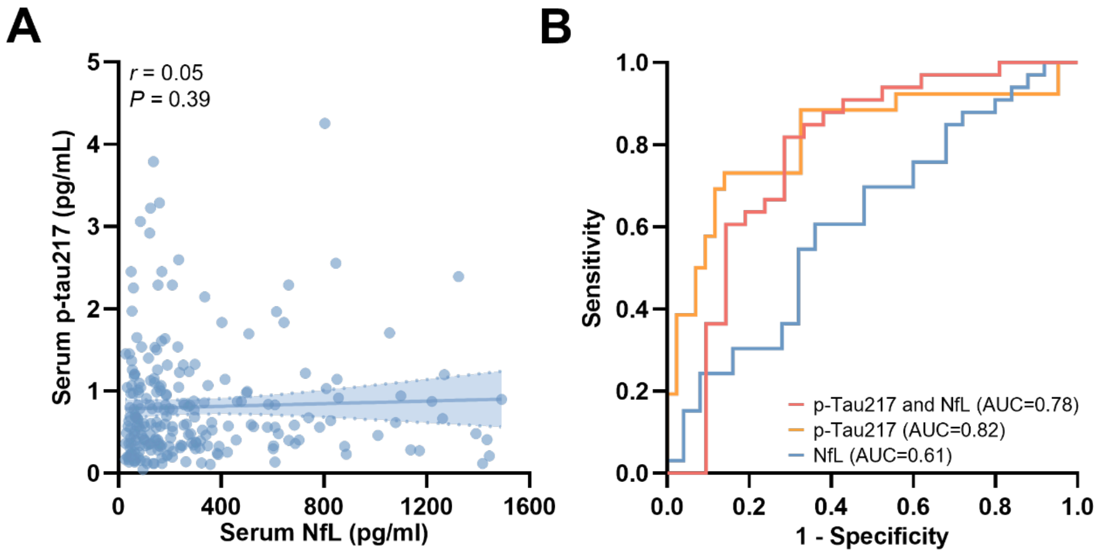

(**A**) Scatter plot showing no significant partial correlation between serum p-tau217 and NfL levels, adjusted for age and sex. (**B**) Logistic regression analysis controlling for age and sex, assessing the performance of p-tau217, NfL, and their combination in distinguishing individuals who phenoconverted to PD-CI or DLB from PD with normal cognition. Least squares regression line with 95% confidence interval is shown. Pearson correlation coefficient and corresponding P value are reported.

Supplementary Figure S8: Kaplan-Meier survival curves for phenoconversion to PD-CI or DLB with subjects stratified by NfL

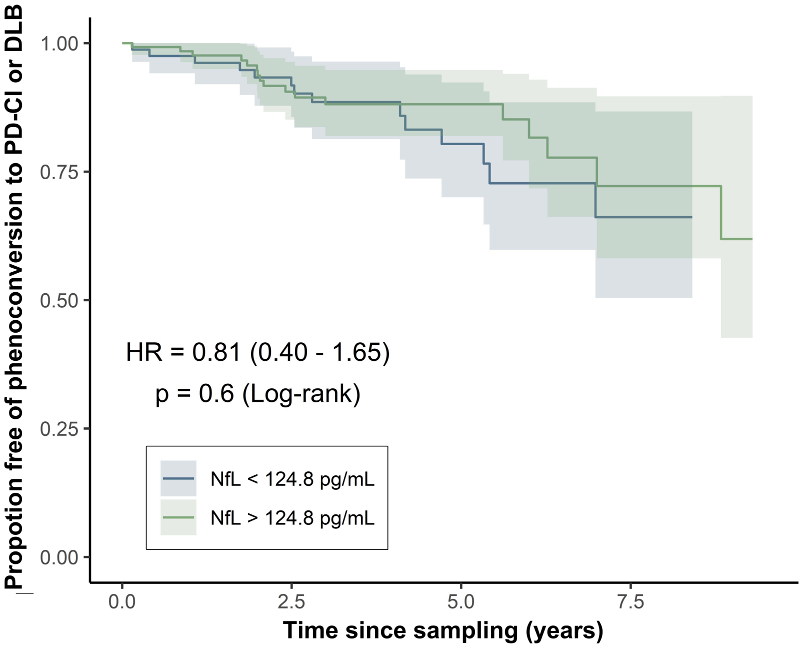

Individuals stratified by NfL levels above or below the optimal threshold (124.8 pg/mL), as determined by the Youden's index for differentiating iRBD individuals with and without CI. The reference group included iRBD individuals who did not phenoconvert or who phenoconverted to other diagnoses. Hazard ratios (HR) with 95% confidence intervals were derived from univariable Cox regression models with patient group as a binary variable.

Supplementary Figure S9: Kaplan-Meier survival curves for phenoconversion to DLB with subjects stratified by p-tau217 using non-phenoconverters without CI as the reference group

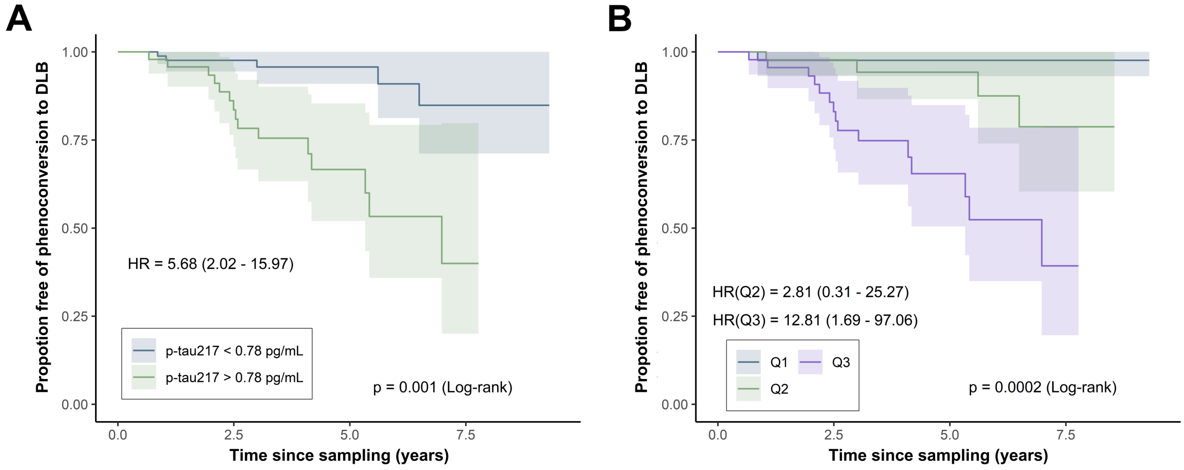

(**A**) Individuals stratified by p-tau217 levels above or below the optimal threshold (0.78 pg/mL) for differentiating iRBD individuals with and without CI. (**B**) Individuals stratified by p-tau217 tertiles (Q1: < 0.405, Q2: 0.405 – 0.810, Q3: > 0.810). Hazard ratios (HR) with 95% confidence intervals were derived from univariable Cox regression models, with patient group as a binary variable (panel **A**) or factor variable with 3 levels (panel **B**).

Supplementary Figure S10: Serum p-tau217 and NfL levels in iRBD stratified by *APOE* status

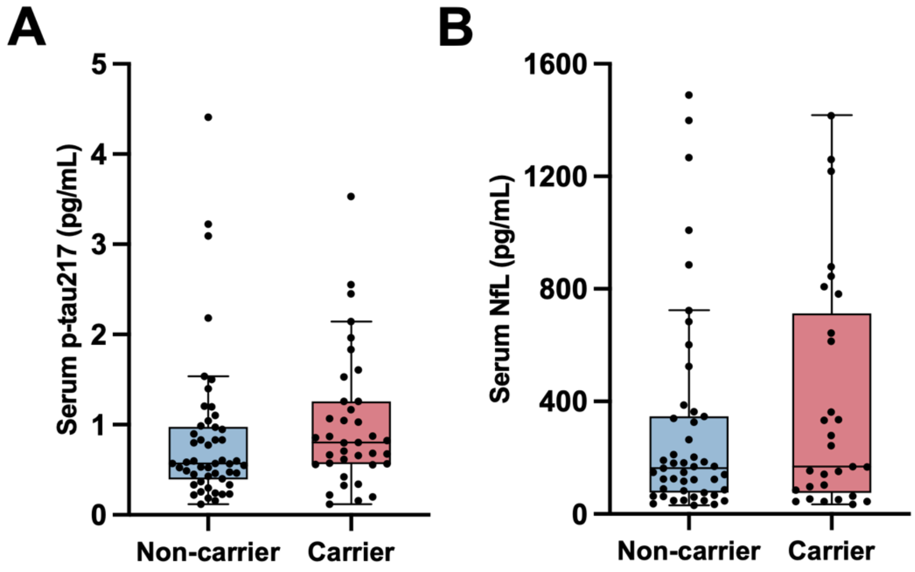

(**A**) *ApoEε4* carriers (*n* = 37) showed slightly higher serum p-tau217 levels (median ± IQR = 0.80 ± 0.69 pg/mL) compared to non-carriers (*n* = 52; median ± IQR = 0.57 ± 0.56 pg/mL) that did not reach statistical significance (Hodges-Lehmann difference = 0.19 pg/ml; 95% CI, −0.01 – 0.39, *P* = 0.07) (**B**) There were no differences in NfL levels between the two groups (Hodges-Lehmann difference = 20.80 pg/ml; 95% CI, −40.28 – 142.26, *P* = 0.52). Boxes represent the interquartile range from the 25th to 75th percentiles, the midline indicates the median, and whiskers and outliers are plotted using the Tukey method. Statistical comparisons were performed using the Mann–Whitney U test.
